# The Photophysical Properties of the Protonation States of SYPRO^®^ Orange in Aqueous Solution

**DOI:** 10.3390/molecules30081691

**Published:** 2025-04-10

**Authors:** Claire E. Baxter, Ali N. Khan, Christina M. Starcevic, Natalie Shkolnik, Jörg Zimmermann

**Affiliations:** Department of Chemistry and Biochemistry, Loyola University Chicago, Chicago, IL 60660, USA; cbaxter3@luc.edu (C.E.B.);

**Keywords:** SYPRO Orange, differential scanning fluorometry, thermal shift assays, coupled deprotonation-aggregation equilibria, fluorescence quenching, molecular aggregates

## Abstract

SYPRO^®^ Orange (SyO) is a zwitterionic dye that is used for protein gel staining, for thermal melt assays of proteins, and as a marker for misfolded proteins. However, while widely utilized, much of SyOs’ photophysics remains unexplored. We studied the effect of pH on the photophysical properties of SyO in aqueous solution and found two well-defined transitions in the 0 to 10 pH range between three SyO species with distinct absorption and fluorescence properties. The first transition occurs around pH 1.5 and appears to be a coupled deprotonation–aggregation event. The second transition occurs between pH 4 and 5, and its pK_a_ depends on the concentration of SyO. A link between the concentration dependence of the pK_a_ of the second pH transition and the aggregation behavior of SyO at neutral pH is discussed, and aggregation equilibrium titrations are presented that suggest that SyO forms multimeric aggregates at neutral pH containing ten or more SyO molecules.

## 1. Introduction

SYPRO^®^ Orange (SyO) is a zwitterionic merocyanine dye that is poorly soluble and has a low fluorescence quantum yield in an aqueous solution at neutral pH. However, when interacting with denatured, partially unfolded, or misfolded protein, a significant increase in the fluorescence intensity of SyO is observed [1,2,3,4,5,6]. SyO is therefore used in various protein applications, most notably as a protein gel stain and in thermal shift assays (TSAs) [3,7,8,9,10]. TSA, also known as differential scanning fluorimetry (DSF), is a versatile high-throughput method for detecting protein–ligand binding [11,12,13]. TSA was originally designed as a qualitative binding assay to screen for early drug candidates, but recent advances have allowed this method to be used to quantitatively measure the equilibrium binding constant (K_D_) for protein–ligand equilibria [13,14,15,16]. Additionally, SyO has been used as a marker for amyloid fibrils and has the potential to aid in the understanding of fibril formation in neurodegenerative diseases [5,6,17,18,19].

Despite its popularity, much of the photophysics of SyO remains unexplored. Most relevant to the use of SyO as reporter dye of denatured or misfolded protein is its behavior in aqueous solutions at neutral pH. There is mounting experimental evidence that under these conditions, SyO has the propensity to form self-aggregates and that the increase in fluorescence intensity upon SyO interacting with denatured or misfolded protein is amplified by de-aggregation [4,5]. However, little is known about the properties of SyO aggregates other than the fact that they form in aqueous solutions at neutral pH and at concentrations above 1× SyO, and that SyO fluorescence is quenched in the aggregated state [4,5].

**Scheme 1:**
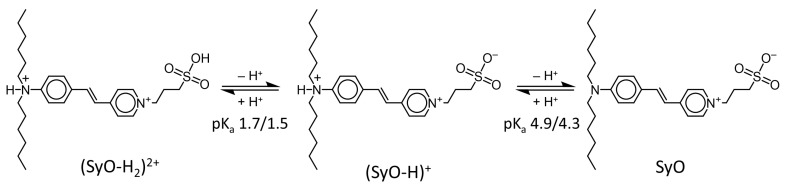
The chemical structure and protonation states of SyO. The pK_a_ values are for 1×/5× SyO concentration (see Table 1).

One open question is the pH dependency of SyO ‘s properties. SyO has two ionizable groups, a sulfonic acid group and a dihexylamine group, located at opposite ends of its styrylpyridine chromophore (Scheme 1). The pK_a_ of the dihexylamine group is of particular interest for the application of SyO since the observed changes in absorbances may be connected to changes in the protonation state of the molecule when associating with protein. Here, we present a systematic study of the effect of pH on the photophysical properties of SyO in aqueous solution. We found two well-defined transitions in the range of pH 0 to pH 10 between SyO species with distinct absorption and fluorescence properties. The first transition occurs around pH 1.5 and appears to be a coupled deprotonation–aggregation event. The second transition occurs between pH 4 and pH 5, and its pK_a_ depends on the concentration of SyO. A link between the concentration dependence of the pK_a_ of the second pH transition and the aggregation behavior of SyO at neutral pH is discussed, and aggregation equilibrium titrations are presented that suggest that SyO forms multimeric aggregates at neutral pH containing ten or more SyO molecules.

## 2. Results and Discussion

### 2.1. pH Titrations

**Figure 1:**
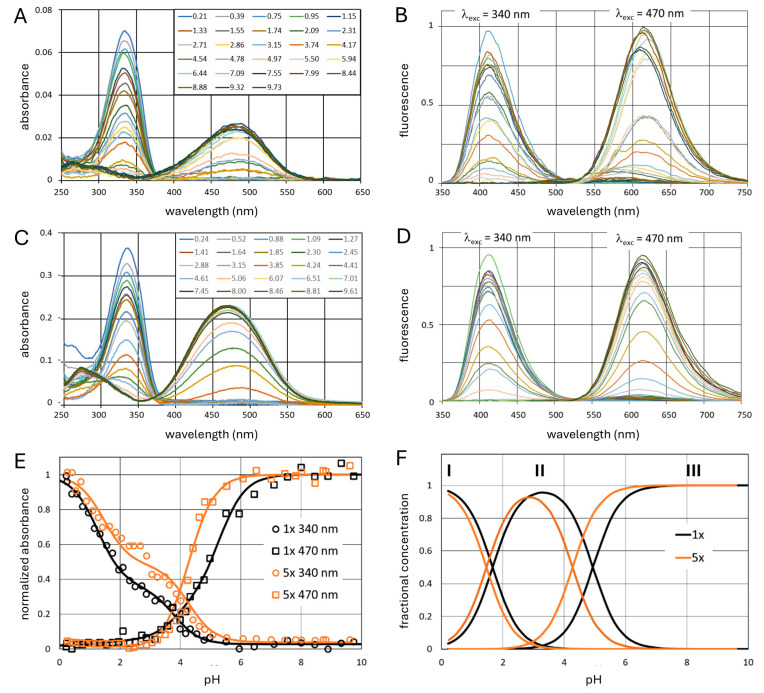
(**A**–**D**) The pH dependence of absorption and fluorescence spectra for 1× (**A**,**B**) and 5× (**C**,**D**) SyO concentrations. The legends in (**A**,**C**) show pH values and apply for both absorption and fluorescence spectra at a given SyO concentration. For fluorescence, the bands for both 340 nm and 470 nm excitation are shown in the same panels. (**E**) The normalized integrated absorptions of the 340 nm band (circles) and 470 nm band (squares) for aqueous solutions of 1× SyO (black) and 5× SyO (orange). The lines are the best fits of the data to a three-state model. The intensities were integrated from 330 to 350 nm and normalized to the low pH values (circles), integrated from 460 to 480 nm, and normalized to the high pH value (squares), respectively. (**F**) Populations of the protonation states of SyO at 1× (black) and 5× (orange) concentrations from the fit of the titration data are shown in panel E for Equations (1) and (2).

Previous work suggests that in aqueous solution at neutral pH, SyO exists as a monomer at concentrations of up to approximately 1× and forms molecular aggregates at higher concentrations [4]. We therefore collected pH titration data at 1× and 5× SyO concentrations to assess the effect of aggregation on the pK_a_s of SyO (Figure 1A–D). At low pH, an absorption band at 340 nm and a fluorescence band at 410 nm were observed for both SyO concentrations, which were replaced by an absorption band at 470 nm and an emission band at 610 nm at high pH. The normalized integrated intensities of the 340 nm and 470 nm absorption bands were found to differ significantly for the two SyO concentrations (Figure 1E). Two deprotonation events were observed, the first being around pH 1, and the second around pH 5 for 1× SyO and pH 4 for 5× SyO. The integrated absorption intensities for the two bands were simultaneously fit to a three-state model (Figure 1E,F; Equations (1) and (2)), yielding the pK_a_ values shown in Table 1.

(1) $$I\left(pH\right)=\sum_{i}a_{i}\;f_{i}(pH)$$

where $a_{i}$ is the normalized intensity, and $f_{i}\left(pH\right)$ are the pH-dependent fractional concentrations of species I to III:

(2) $$f_{I}\left(pH\right)\;=A_{0}/\left(1+K_{a1}{\left[H_{3}O^{+}\right]}^{-1}+K_{a1}K_{a2}{\left[H_{3}O^{+}\right]}^{-2}\right)\;f_{II}\left(pH\right)\;=K_{a1}\;f_{I}\left(pH\right)/\left[H_{3}O^{+}\right]\;{\begin{array}{c} f \end{array}}_{III}(pH)=K_{a1}K_{a2}\;f_{I}\left(pH\right)/{\left[H_{3}O^{+}\right]}^{2}$$

where $A_{0}$ is the total concentration of the diprotic acid, and $K_{a1}$ and $K_{a2}$ are the acid dissociation constants for the first and second deprotonation equilibriums.

**Table 1:** pK_a_ values from fit of normalized absorption data (Figure 1E).

Transition	${\mathrm{pK}}_{a}\;(1\times \mathrm{SyO})$	${\mathrm{pK}}_{a}\;(5\times \mathrm{SyO})$
state I/II	1.7	1.5
state II/III	4.9	4.3

Using the pK_a_ values obtained from fitting the integrated absorption data, we next determined the protonation state-associated spectra (PSAS) for species I to III by fitting the pH-dependent absorption and fluorescence spectra, $I\left(pH,\lambda \right)$, to

(3) $$I\left(pH,\lambda \right)=\sum_{i}c_{i}\left(pH\right)\;PSAS_{i}\left(\lambda \right)$$

where $c_{i}(pH)$ are the fractional concentrations of species I to III, and $PSAS_{i}(\lambda )$ are the PSAS for the different species. The PSAS were determined by fitting the experimentally observed $I\left(pH,\lambda \right)$ separately for each wavelength using the fractional concentrations $c_{i}(pH)$ determined from the fit of the integrated absorption intensities (Figure 1F).

The PSASs of species I and II are limited to the blue-shifted absorption (340 nm) and fluorescence (410 nm) bands, while the PSASs of species III are limited to the red-shifted absorption (460 nm) and fluorescence (610 nm) bands. There is virtually no “bleeding” of the blue-shifted bands into the PSASs of species III or red-shifted bands into the PSASs of species I and II, confirming that the large red shift for both absorption and fluorescence upon increasing pH occurs for the transition from state II to state III and that only three species are needed to fit the pH-dependent spectra of SyO between pH 0 and 10. The large spectral shifts between states I/II and state III are most likely due to the deprotonation of the dihexylamine that is adjacent to the π electron system of the styrylpyridine chromophore rather than the deprotonation of the sulfonic acid group, which is separated from the chromophore by three saturated carbons and is expected to have a pK_a_ value well below zero [20]. Interestingly, the pK_a_ value of the transition between states II and III decreases by 0.6 pH units with an increasing SyO concentration. This suggests that state III is stabilized at a higher SyO concentration, likely due to the aggregation of SyO in state III, as suggested previously [4]. In contrast, the PSAs of states I and II have virtually identical spectral shapes and only differ in intensity, and the pK_a_ value of the transition between states I and II changes little between 1× and 5× SyO.

**Figure 2:**
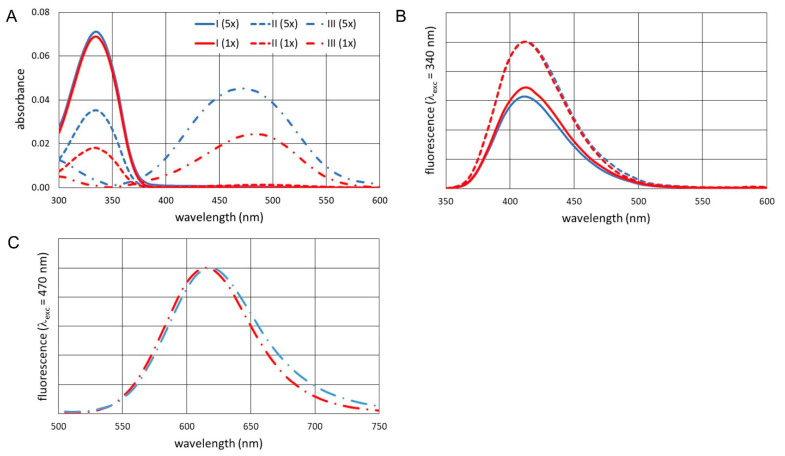
PSAS for (**A**) absorption normalized to 1x SyO, (**B**) fluorescence for $\lambda_{exc}$ = 340 nm normalized to PSAS of state II, and (**C**) fluorescence for $\lambda_{exc}$ = 470 nm normalized to PSAS of state III (red curves—1× SP; blue curves—5× SyO; solid lines—state I; dashed lines—state II; dashed–dotted lines—state III).

The PSASs of state I are independent of the SyO concentration, while the PSASs of states II and III show clear concentration dependencies, most notably an approximately two-fold difference in normalized absorbance for 1× and 5× SyO that was observed for both states II and III (Figure 2A), and a slight red shift in the state III PSAS at the higher SyO concentration (Figure 2C).

Since we assigned the transition between states II and III to the deprotonation of the dihexylamine, the obvious choice for the deprotonation event that is associated with the transition between states I and II would be the deprotonation of the sulfonic acid group. However, as mentioned above, the pK_a_ values of sulfonic acids are usually well below zero, and it seems unusual for the pK_a_ of a sulfonic acid group to be up-shifted to positive values. A mechanism that would explain pK_a_ shifts is the aggregation of SyO in state I, which would stabilize state I relative to state II and shift the pK_a_ of the transition to higher pH values, but as will be shown in the next section, there is no evidence for the aggregation of SyO in state I at concentrations below 9× SyO.

### 2.2. Aggregation Behavior of States I, II, and III Was Observed

**Figure 3:**
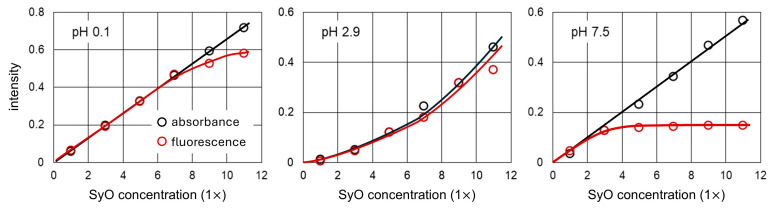
Absorption and fluorescence intensity as function of SyO concentration at three different pH values corresponding to maximum population of states I (pH 0.1), II (pH 2.9) and III (pH 7.5). Shown are absorbance at 340 nm and fluorescence at 410 nm (340 nm excitation) for pH 0.1 and 2.9, and absorbance at 470 nm and fluorescence at 620 nm (470 nm excitation) for pH 7.5. Intensity values on y axis correspond to absorbance of sample in 1 cm cuvette. Fluorescence intensities are scaled to match initial slope of absorption intensities. Lines are not fitted but are meant to guide viewer’s eye.

To test for evidence of molecular aggregation in states I, II, and III, we determined the concentration dependencies of SyO absorption and fluorescence at pH values for which the concentration of a given state is at its maximum (pH 0.1 for state I, pH 2.9 for state II, and pH 7.5 for state III). The respective titration curves are shown in Figure 3.

As is apparent from the figure, state I does not show signs of aggregation below a SyO concentration of 9× since both absorption and fluorescence intensities change linearly with the SyO concentration. For state II, both absorption and fluorescence change nonlinearly with the SyO concentration but remain proportional to each other except for the data point at 11× SyO. The most pronounced misalignment between absorption and fluorescence intensities was observed for state III, for which absorbance is proportional to the SyO concentration over the entire concentration range, but fluorescence plateaus for concentrations larger than 3×. We note that a substantial deviation from linearity has been reported previously for SyO fluorescence in an aqueous solution at neutral pH [4]. However, we could not reproduce the observations found in Ref. [4], which show that the fluorescence intensity decreases for concentrations above 1× SyO at pH 7.5. Rather, we observed that the fluorescence intensity plateaus above 1× SyO. This discrepancy is likely due to the different experimental setup in Ref. [4], which used a plate reader with a bandpass filter that recorded the integrated fluorescence between 465 nm and 580 nm and therefore only included the blue edge of SyO fluorescence, while we analyzed the entire spectrum. Nevertheless, we came to the same conclusion as Ref. [4], that is, SyO forms molecular aggregates at neutral pH in which fluorescence is significantly quenched. Such aggregation-induced quenching has been widely studied [21,22,23], and several mechanisms have been suggested to explain the phenomenon, e.g., excimer formation or molecular rotation [24,25,26,27]. It may be surprising to find a linear relationship between concentration and absorbance in state III even when molecular aggregation occurs, as evidenced by fluorescence quenching. This may be explained by weak electronic coupling between the molecular transition dipoles in the aggregates, in which case the decrease in concentration from monomer to aggregate is compensated by an approximately proportional increase in the aggregate’s transition dipole [28].

### 2.3. Properties of the Molecular Aggregates Formed in States II and III

The absorption of the state II aggregates at 340 nm increases nonlinearly with an increasing SyO concentration (Figure 3, pH 2.9), suggesting that state II aggregates have a larger molar extinction per molecule than the monomer. This behavior may be a sign of “super-additivity” linked to the formation of J-aggregates [29]. However, “super-additivity” is usually accompanied by spectral narrowing and red-shifting of the corresponding absorption band, neither of which is observed in the absorption spectra of 5× SyO. However, the increase in absorption per molecule is moderate (two-fold for 5× compared to 1× SyO), whereas J-aggregates usually show a significantly larger increase in absorption intensity. Therefore, one may still speculate that species II forms “head-to-tail” J-aggregates, perhaps facilitated by the zwitterionic nature of species II with a negatively charged sulfonic acid group on one end and a positively charged protonated dihexylamine on the other end of the linear molecule.

**Figure 4:**
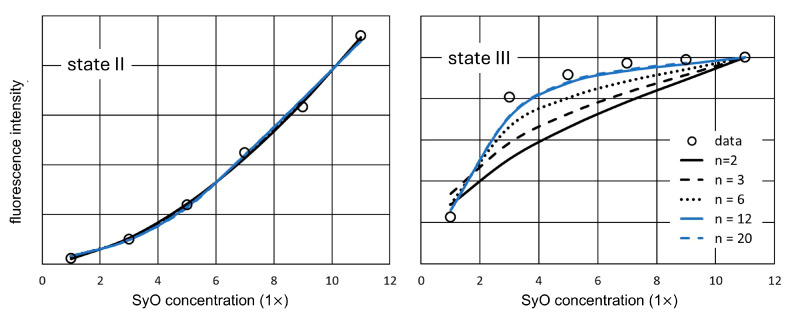
The best fit of a two-state aggregation equilibrium (Equation (4)) to the experimentally observed concentration-dependent fluorescence intensities of states II and III (circles) from Figure 3 for different aggregate sizes n (legend, right panel). Both the equilibrium constants and fluorescence quantum yields of the monomer and aggregate were free fit parameters. (State II: 340 nm excitation, 410 nm emission; state III: 470 nm excitation, 620 nm emission).

To estimate aggregate size, we fit the concentration-dependent fluorescence intensities for states II and III to a two-state equilibrium between monomer M(aq) and the n-mer aggregate ${\left(M\right)}_{n}\left(aq\right)$:

(4) $$n\;M(aq)\overset{\;{\;K}_{n\;}\;}{\to }\;{\left(M\right)}_{n}\left(aq\right)$$

where $K_{n}$ is the equilibrium constant for aggregation. Experimental data were fit by first numerically solving the equilibrium expressions for each SyO concentration for a given $K_{n}$, and second, to carry out least-square fitting of the experimental data using the monomer and aggregate concentrations from the previous step and varying the fluorescence quantum yields of the monomer and aggregate. $K_{n}$ was varied to find the global minimum in $\chi^{2}$ for the second step, and the resulting fits are shown in Figure 4 for various aggregate sizes n.

The fit is inconclusive for state II since all aggregate sizes give fits of similar quality. However, for state III, the fit suggests an aggregate size of n=10 or more because fits with smaller aggregate sizes cannot reproduce experimental data.

Shifts in the apparent pK_a_ due to the aggregation of a protonated or deprotonated species can be rationalized with the following simplified coupled reaction equations:

(5) $$HA\left(aq\right)+H_{2}O(l)\overset{K_{a}}{\to }A^{-}\left(aq\right)+H_{3}O^{+}(aq)\;n_{A^{-}}A^{-}(aq)\overset{K_{n,A^{-}}}{\to }{{(A}^{-})}_{n_{A^{-}}}\left(aq\right)\;n_{HA}HA(aq)\overset{K_{n,HA}}{\to }{\left(HA\right)}_{n_{HA}}\left(aq\right)$$

where HA(aq) and $A^{-}(aq)$ are the acid and conjugate bases undergoing the pH transition, $K_{a}$ is the acid dissociation constant of HA(aq), and ${{(A}^{-})}_{n_{A^{-}}}\left(aq\right)$ and ${\left(HA\right)}_{n_{HA}}$ are the molecular aggregates formed by the acid and conjugate base with the monomer–aggregate equilibrium constants $K_{n,A^{-}}$ and $K_{n,HA}$, respectively. At high SyO concentrations, the experimentally observed pH transition will occur between the aggregated acid and the aggregated conjugate base:

(6) $$\;\frac{1}{n_{HA}}\;{\left(HA\right)}_{n_{HA}}\left(aq\right)+H_{2}O\left(l\right)\;\overset{\;K_{a}^{\prime }\;}{\to }\;\frac{1}{n_{A^{-}}}{\left(A^{-}\right)}_{n}\left(aq\right)+H_{3}O^{+}(aq)$$

for which the apparent equilibrium constant, pK_a_’, can be expressed as follows:

(7) $$pK_{a}^{\prime }\approx pK_{a}+\frac{1}{n_{HA}}\log K_{n,HA}-\frac{1}{n_{n_{A^{-}}}}\log K_{{n,A}^{-}}$$

In agreement with Le Chatelier’s principle, the apparent pK_a_’ thus shifts toward values larger than the monomeric pK_a_ if $K_{n,HA}>1$ and toward lower values if $K_{n,A^{-}}>1$. Therefore, comparing the pK_a_ determined under conditions where aggregates are not formed (here, 1× SyO) with the apparent pK_a_’ determined under conditions where aggregates do form (here, 5× SyO) allowed us to estimate the equilibrium constants for aggregate formation.

We observed a decrease in the apparent pK_a_ of 0.2 and 0.6 for the transition between states I and II and between states II and III, respectively, when the SyO concentration increased from 1× to 5× (Table 1). Experimental evidence suggests that state I is monomeric at both concentrations (the normalized PSAS are identical for both concentrations, as shown in Figure 2A,B, and the absorption and fluorescence intensities change linearly over the concentration range, as shown in Figure 3). Therefore, the decrease in the apparent pK_a_ is solely due to the aggregation of SyO in state II. The fairly small change in the apparent pK_a_ suggests a moderate $K_{n}$ in state II but could also be the result of partial aggregation.

For the transition between states II and III, a decrease in the apparent pK_a_ of 0.6 pH units was observed. For this transition, the state II aggregate acts as acid, therefore causing an increase in the apparent pK_a_ by 0.2 pH units. It follows that the state III aggregate causes the apparent pK_a_ to decrease by 0.8 pH units, resulting in the observed net decrease of 0.6 pH units. This would place $K_{n}$ for the state III aggregate in the range of $10^{8}$ for n=10.

**Figure 5:**
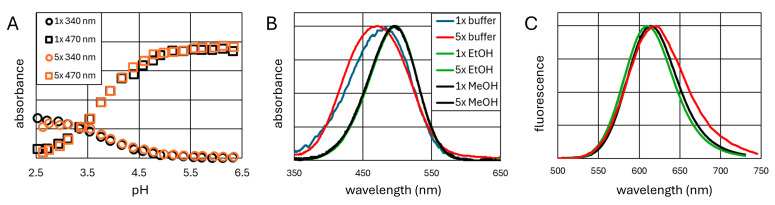
(**A**) Integrated absorptions of the 340 nm band (circles) and 470 nm band (squares) for aqueous solutions of 1× SyO (black) and 5× SyO (orange). The absorbances of the 5× solutions were divided by a factor of 5 to normalize for concentration. (**B**,**C**) Normalized absorbance (**B**) and fluorescence (**C**) spectra for 1× and 5× SyO in phosphate buffer, pH 7.5, EtOH, and MeOH.

To further support our conclusion that molecular aggregation in states II and III lead to the apparent shift in the pK_a_, at a 5× SyO concentration, we performed a titration in the presence of 0.2% sodium dodecyl sulfate (SDS). SDS forms micelles at this concentration, and due to its amphiphilic nature, SyO may be expected to be absorbed by SDS micelles rather than forming molecular aggregates in an aqueous environment. Indeed, the titration curves around the transition between states II and III are virtually identical at 1× and 5× SyO concentrations in the presence of SDS (Figure 5A), suggesting that SyO is indeed associated with SDS micelles. Interestingly, the pK_a_ of SyO in SDS micelles is significantly downshifted, likely due to the stabilization of state III in the micelles. We also collected the spectra of SyO dissolved in EtOH and MeOH for which SyO is expected to exist in monomeric form [5]. Normalized absorption and emission spectra are virtually identical in EtOH and MeOH, while the corresponding spectra in aqueous solution at pH 7.5 are concentration dependent and significantly blue-shifted compared to the spectra in EtOH and MeOH for absorption but not fluorescence (Figure 5B,C), which is in agreement with the formation of molecular aggregates with quenched fluorescence in state III.

## 3. Methods

The concentration of SyO stock solutions is provided by the manufacturer in units of 1× SyO (SyO is offered as a 5000× stock solution). We will thus report SyO concentrations in units of 1× SyO but note that according to several sources, 1× SyO corresponds to a concentration of 2 μM [14,30]. SyO was purchased from ThermoFisher as a 5000× stock solution in dimethyl sulfoxide (DMSO). The 5000× solution was diluted in ultrapure water to a concentration of 20×. The resulting solution, which was used as stock solution for pH titrations, thus contained 0.4% (*v*/*v*) DMSO. All further dilutions maintained the 0.4% (*v*/*v*) DMSO concentration by adding appropriate amounts of DMSO (HPLC grade, Sigma) to the aqueous solvents. The following buffers were used in pH titrations: pH 2.2 to 6.5, 10 mM citrate buffer; pH 7 to 9, 10 mM tris(hydroxymethyl)aminomethane (Tris) buffer; and pH 9.5 to 11, 10 mM N-cyclohexyl-3-aminopropanesulfonate (CAPS) buffer. Due to concerns that the size of citrate ions may influence the molecular aggregation behavior of SyO, control experiments were performed in unbuffered HCl solutions at pH 2.9. No differences in the behavior of SyO in unbuffered solutions and citrate-buffered solutions were found. For pH values less than 2, the pH was adjusted by adding the appropriate amount of hydrochloric acid. As described above, each buffer also contained 0.4% DMSO. The pH of each solution was measured with a calibrated pH meter immediately before data collection. Absorption spectra were measured with a double-beam instrument (Shimadzu UV-2450), and fluorescence spectra were measured with a JASCO-8300 fluorometer. All spectroscopic data were collected at ambient temperature in 1 cm quartz cuvettes. Data were analyzed in Microsoft Excel, and least-square fitting was carried out with a custom-created code in Matlab (MathWorks Inc., Natick, MA, USA). The Equations (1) and (2) was used to fit the normalized integrated absorption intensities I(pH).

## 4. Conclusions

We investigated the pH-dependent photophysical properties of SyO. In the pH range of 0 to 10, two deprotonation events were found. The first transition occurred with a pK_a_ of 1.5 and was assigned to the deprotonation of the sulfonic acid group of SyO. Sulfonic acid groups usually have a pK_a_ well below zero, and the reason for the unusually high pK_a_ in SyO requires further investigation. The second transition was accompanied by a dramatic red-shift in both the absorption and fluorescence spectra, and the apparent pK_a_ of the transition changed with the SyO concentration. At a 1× SyO concentration, its pK_a_ was 4.9, while at a 5× SyO concentration, it was 4.3. We assigned this transition to the deprotonation of the dihexylamine group of SyO. In agreement with previous reports, we found that the high-pH species forms molecular aggregates. Fits of the concentration-dependent fluorescence intensity suggests that these aggregates contain ten or more SyO molecules. The intermediate species also appeared to form molecular aggregates, but they were less stable than the aggregates of the high-pH species, which explains the decrease in the apparent pK_a_ of the second transition with an increasing concentration of SyO.

## Data Availability

Raw datasets are available in the Figshare data repository (https://doi.org/10.6084/m9.figshare.c.7756232).

## Abbreviations

DMSO: dimethyl sulfoxide; DSF, differential scanning fluorometry; PSAS, protonation state-associated spectrum; SyO, SYPRO^®^ Orange; TSA, thermal shift assay.
